# Predictability of maxillary positioning: a 3D comparison of virtual and conventional orthognathic surgery planning

**DOI:** 10.1186/s13005-021-00279-x

**Published:** 2021-07-13

**Authors:** Anja Quast, Petra Santander, Timon Kahlmeier, Norman Moser, Henning Schliephake, Philipp Meyer-Marcotty

**Affiliations:** 1 Department of Orthodontics, University Medical Center Goettingen, Robert-Koch-Str. 40, 37075 Göttingen, Germany; 2 Department of Oral and Maxillofacial Surgery, University Medical Center Goettingen, Robert-Koch-Str. 40, 37075 Göttingen, Germany

**Keywords:** Orthognathic surgery, Orthodontic-surgical treatment, Osteotomy, BSSO, Le Fort I, Accuracy, Maxillary positioning

## Abstract

**Background:**

Virtual surgery planning (VSP) is believed to reduce inaccuracies in maxillary positioning compared to conventional surgery planning (CSP) due to the elimination of face-bow transfer and laboratory steps. However, there is still a lack of comparative studies for the accuracy of splint-based maxillary positioning in CSP versus VSP. Therefore, the objective of this retrospective, observational study was to compare if splints produced by VSP and CSP reach postoperative outcomes within clinically acceptable limits.

**Methods:**

The planned and actual postoperative results of 52 patients (VSP: n = 26; CSP: n = 26) with a mean age of 24.4 ± 6.2 years were investigated by three-dimensional (3D) alignment with planning software. The conventional treatment plan was digitized, so that the evaluation of both methods was performed in the same manner using the same coordinate system. Inaccuracies were measured by sagittal, vertical and transversal deviations of the upper central incisors and the inclination of the maxillary occlusal plane between the planned and achieved maxillary positions.

**Results:**

Both methods demonstrated significant differences between the planned and actual outcome. The highest inaccuracies were observed in vertical impaction and midline correction. No significant differences between CSP and VSP were observed in any dimension. Errors in vertical and sagittal dimension intensified each other.

**Conclusions:**

In conclusion, splint-based surgeries reached similar results regardless of the applied planning method and splint production.

## Background

In orthognathic surgery, virtual planning is gradually taking over in clinical practice. The possibilities range from CAD/CAM manufactured splints to customized titanium plates as cutting guides or splint-less navigation [1, 2]. Besides the improved visualization of craniofacial deformities, such as occlusal canting and asymmetries [3, 4], virtual surgery planning (VSP) is believed to be less time-consuming and less expensive than conventional surgery planning (CSP) [5]. The elimination of face-bow transfer and the mounting of dental casts, which are known as major sources of error in CSP [6–8], provide further arguments for the use of VSP.

In articulator experiments, rapid-prototyped surgical splints and manually manufactured splints showed similar accuracy [9, 10]. However, the error expected by the laboratory steps in CSP is less based on splint manufacturing itself but more on the transfer of the patient’s individual inclination of the occlusal plane and the orientation of the plaster casts mounted on the articulator [7, 11]. For example, an angular discrepancy between the real and the mounted occlusal plane of 20° may lead to a vertical maxillary displacement of more than 3 mm during a planned sagittal forward movement of 10 mm [11]. This type of error is avoided in VSP.

Several studies have investigated the accuracy and predictability of either conventional [12–15] or virtual planning procedures [1, 16–18]. However, only two studies compared both methods within the same team of clinicians in rather small samples [19, 20]. Their analyses were based on linear measurements in CTs [19] or two-dimensional (2D) measurements in cephalograms [21]. The advantages of three-dimensional (3D) imaging have not been fully used thus far. Therefore, there is still a lack of comparative studies for the accuracy of splint-based maxillary positioning in CSP versus VSP [22].

The knowledge of errors in maxillary positioning is essential as the patients usually have high expectations about this elective surgery [23] and are known to be very sensitive to post-surgical facial deviations [24]. In general, positional differences within 2 mm are assumed as clinically irrelevant [17, 19, 20]. For midline deviations, a more stringent threshold of 1 mm should be applied [25]. The aim of the present study was to investigate if the differences between the planned and achieved postoperative outcomes are within these clinically acceptable limits in splint-based VSP and CSP, and to compare whether one method is superior to the other.

## Methods

This retrospective, comparative study was approved by the Institutional Ethics Committee (no. 7/1/16) in accordance with the Declaration of Helsinki. All patients provided written informed consent to participate in the study.

### Patients

The subjects were 52 healthy adult patients (CSP: *n* = 26; VSP: *n* = 26) with pronounced malocclusions and indications for combined orthodontic-surgical treatment, who were categorized as > grade 4 according to the Index of Orthognathic Functional Treatment Need [26]. The sample size of 52 subjects (26 per group) was determined with G * Power (v. 3.1.9.2, University of Düsseldorf) by applying a significance level of 0.05, a power of 0.8 and a large effect size of 0.8. The effect size was estimated for a clinically relevant mean linear difference between the planned and the postoperative position of 1 mm [20].

All patients underwent orthognathic surgery planning at the Department of Orthodontics and underwent surgery under the supervision of the fourth author (N.M.) at the Department of Oral and Maxillofacial Surgery at the University Medical Center Goettingen between 2016 and 2019. Inclusion criteria were as follows: (1) Le Fort I or bimaxillary osteotomy, (2) availability of pre- and postoperative cone beam computed tomography (CBCT), and (3) availability of planning records. Patients with cleft lip and palate, craniofacial syndromes or isolated BSSO were excluded from the study. All patients were recruited consecutively from our records.

### Surgery planning

For the fabrication of dental plaster casts, alginate impressions of the upper and lower jaw (Tetrachrom, Kaniedenta, Herford, Germany) were taken 4 to 8 weeks preoperatively. The centric relation of the mandible was recorded, and preoperative CBCT scans (PaX Zenith 3D, OrangeDental, Biberach an der Riss, Germany; field of view 240 × 190 mm, voxel size 0.3 mm) were performed with this record in situ to maintain the centric position. Postoperative CBCT scans were taken within 2 weeks after surgery while the final splint kept the mandible in the desired postoperative position. No active orthodontic treatment occurred during this period.

All records came from a period when VSP was implemented in a two-step process in our clinic, so that the timing of the surgery was decisive for group allocation. In the first step, 3D imaging and cephalometry were introduced, but the maxillary displacement and splint production were performed conventional (= CSP group). In the second step, surgery planning was fully virtual including 3D printing of the splints (= VSP group). Therefore, both groups benefited from 3D diagnostics. Patients of the CSP group were operated prior to the patients of the VSP group.

### Virtual surgery planning (VSP) group

VSP was performed in ProPlan CMF (Materialise, Leuven, Belgium). The dental casts were digitized twice by structured-light scanning (S300 Ortho Scanner Zirkonzahn, Gais, Italy), first in final occlusion and second for each jaw individually. Final occlusion was determined by maximum intercuspidal contact and physiological overjet and overbite. For the creation of a 3D head model, the scanned dental casts were aligned with the Digital Imaging and Communications in Medicine (DICOM) data from the CBCT scan. Virtual osteotomies were executed and the maxillomandibular complex was moved into its postoperative position according to the treatment plan. A virtual intermediate splint was designed between the maxilla in the postoperative position and the mandibula in the preoperative position. The virtual splints were exported as.stl files and fabricated with 3D stereolithography (SLA) printing (Form 2, Formlabs, Somerville, Massachusetts, USA) using a biocompatible, photopolymer resin (Dental SG, Formlabs, Somerville, Massachusetts, USA; flexural strength post-cured: > 50 MPa). The virtual treatment plan was stored in the program and was available for subsequent comparison with the postoperatively achieved outcome.

### Conventional surgery planning (CSP) group

For CSP, the dental casts were mounted into an articulator (SAM 3, SAM Präzisionstechnik, Gauting, Germany) with the help of face-bow transfer according to a previous report [7]. Surgery planning was performed using the model positioning device (SAM Präzisionstechnik, Gauting, Germany) as described in detail elsewhere [27]. Clinical photographs and the 3D information from the CBCT scan were available to determine the final position of the dental casts. Three sets of dental casts of the upper and the lower jaw were used for each patient with bimaxillary osteotomy: (a) upper and lower jaw in the preoperative, centric position; (b) upper jaw in the postoperative and lower jaw in the preoperative, centric position; and (c) upper and lower jaw in the postoperative position. For patients who underwent isolated Le Fort I osteotomy, only dental cast sets (a) and (b) existed. An intermediate splint was fabricated on dental cast set (b), i.e., between the desired postoperative maxillary position and the preoperative mandibular position (Weitur Press Standard, Johannes Weithas GmbH & Co.KG, Lütjenburg, Germany; flexural strength post-cured: 78 MPa).

### Surgery protocol

The surgery protocols were the same for all patients: Prior to Le Fort I osteotomy, the condyles were repositioned using the previously described splint-method [28]. In bimaxillary surgeries, maxillary osteotomy was performed first according to a well-recognized protocol [29]. The intermediate splint was used to place the maxilla in the desired position. Sagittal and transversal maxillary positions were completely determined by the surgical splint. The vertical position was adjusted using internal reference points as described by Schwestka-Polly [27]: the surgeon measured the distance between a fixed point in the lower jaw, e.g. an orthodontic bracket or a drilled hole, and a fixed point in the upper jaw above the osteotomy line using a divider. If this distance was constant before Le Fort I and after Le Fort I with the intermediate splint inserted, the surgeon assumed to have reached the planned vertical position.

### Comparison of planned and actually achieved maxillary positioning

**Fig. 1:**
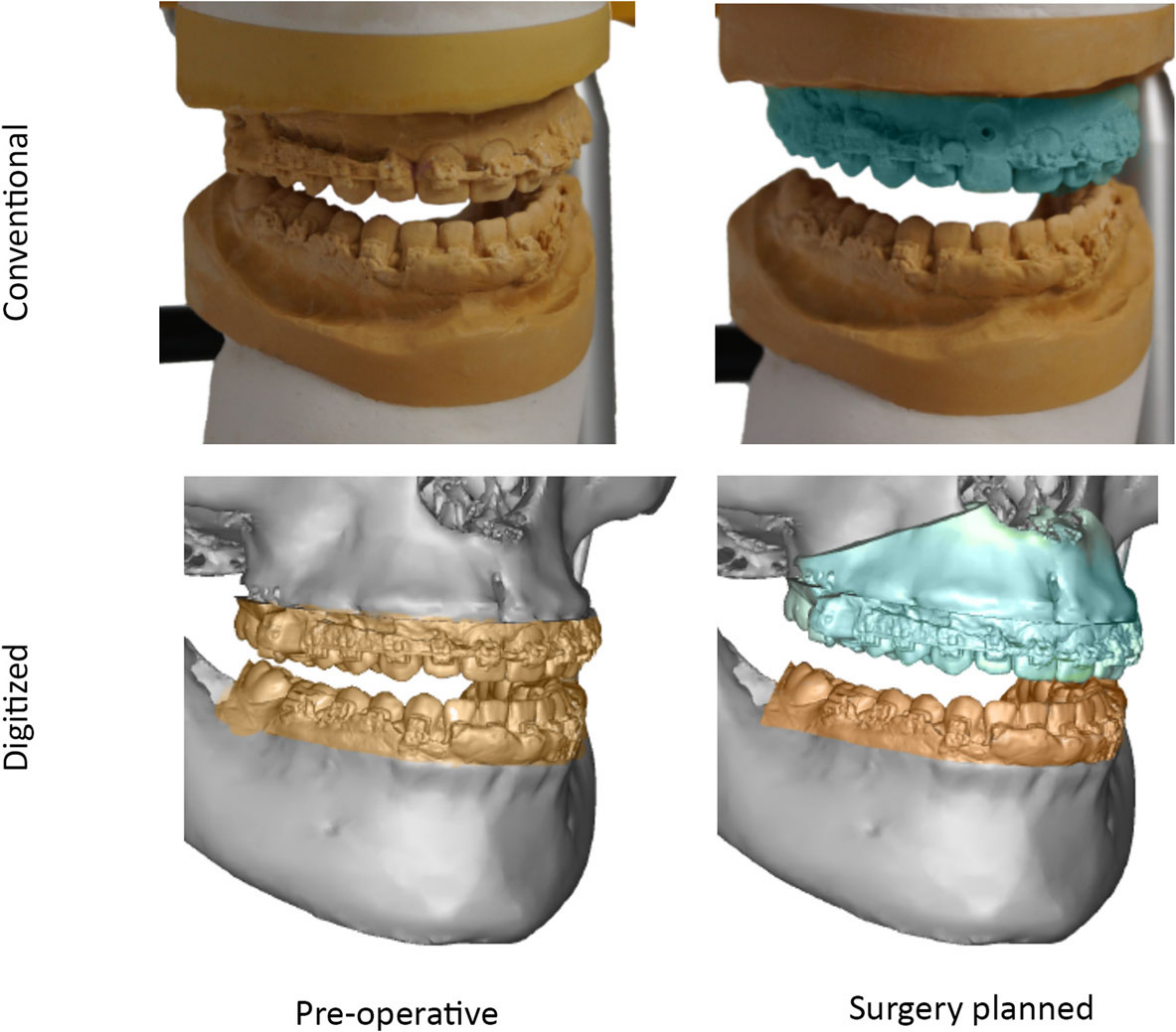
Digital reconstruction of the conventional surgery planning: Left side: Alignment of the preoperative CBCT scan and the dental casts with the upper and lower jaw in the preoperative, centric position. Right side: Reconstruction of the planned postoperative maxillary position by alignment of the preoperative mandible and the osteotomized maxilla as bony structures and the upper jaw in the postoperative and lower jaw in the preoperative position as dental casts. The mandible served as fixed reference while the maxilla moved in the planned postoperative position

To compare the planned and achieved maxillary positions for both planning methods in the same software with the same coordinate system, the CSP had to be digitized: dental cast sets (a) with the upper and lower jaw in the preoperative, centric position and (b) with the upper jaw in the postoperative and lower jaw in the preoperative, centric position were structured-light scanned. A 3D head model was created by aligning the preoperative CBCT scan and the preoperative dental cast set (a). To achieve the digitized postoperative maxillary position, the maxilla was osteotomized while the mandible was kept as reference in the centric position and the lower jaw of dental cast set (b) was superimposed with this reference. The upper jaw of dental cast set (b) was used to move the osteotomized maxilla in the conventionally planned position (Fig. 1). As the dental casts kept their relationship to each other during structured-light scanning, this method allowed virtual re-creation of CSP. The error of superimposing the dental casts on the CBCT scan was assumed to be the same as for the process in VSP. This error was previously assessed for our clinical setting and had an average root mean square of 0.28 ± 0.04 mm.

To compare the planned and actual outcomes, the postoperative CBCT was used to create a 3D head model. To improve the image quality at the dental level, the scanned casts were aligned with the CBCT. The head models of the preoperative treatment plan were then superimposed with the postoperative head models by an iterative closest point algorithm (number of iterations: 10; subsample percentage 15%; final distance threshold: 0.3 mm) in the software 3-matic Research (Materialise NV, Leuven, Belgien). The mean error of this alignment method was 0.22 ± 0.08 mm. By this method, we were able to compare the planned and achieved maxillary positions in the same data set and the same coordinate system.

The accuracy of maxillary positioning was evaluated in all three dimensions in ProPlan CMF. To measure distances and angulations, a cartesian coordinate system was established based on the midsagittal (x-axis), the frontal (y-axis) and the Frankfurt Horizontal plane (z-axis). Translational discrepancies between the planned and the achieved maxillary position were assessed by evaluating the difference of the maxillary dental midline between both positions. The rotational movements of the maxilla were described by the discrepancy in inclination of the occlusal plane projected onto the frontal, the midsagittal and the Frankfurt Horizontal plane (see Table 1 for detailed description). The discrepancies between the planned and achieved maxillary positions were calculated as the mean and absolute difference. The mean differences considered whether the maxillary position was over- or undercorrected with positive values for undercorrection and negative values for overcorrection. The absolute differences revealed the total discrepancy regardless of the direction of the malpositioning.

**Table 1:** Definition of the translational and rotational discrepancies between the planned and the achieved maxillary position

Abbreviation	Measurement	Definition
Translation—linear measurements (mm)		
U1 (x)	Sagittal translation (x-axis)	Shortest distance between the midpoint of the upper central incisors on the planned and the achieved maxillary position in sagittal direction
U1 (y)	Transversal translation (y-axis)	Shortest distance between the midpoint of the upper central incisors on the planned and the achieved maxillary position in transversal direction
U1 (z)	Vertical translation (z-axis)	Shortest distance between the midpoint of the upper central incisors on the planned and the achieved maxillary position in vertical direction
Rotation—Angular measurements (°)		
OcP (x)	Rotation of the upper occlusal plane around the sagittal (x-) axis	Angular difference between the upper occlusal plane of the planned and the achieved maxillary position projected onto the frontal plane
OcP (y)	Rotation of the upper occlusal plane around the transversal (y-) axis	Angular difference between the upper occlusal plane of the planned and the achieved maxillary position projected onto the midsagittal plane
OcP (z)	Rotation of the upper occlusal plane around the vertical (z-) axis	Angular difference between the maxillary sagittal plane of the planned and the achieved maxillary position projected onto the Frankfurt Horizontal plane

Translational differences smaller than 2 mm and rotational differences smaller than 4° were assumed to be clinically insignificant. For midline deviations, a stricter threshold of 1 mm was applied [17, 25].

All measurements were performed by the same examiner (T.K.). To assess intrarater agreement, the examiner repeated the measurements on 10 randomly selected patients on a second occasion more than 6 months later. For interrater agreement, a second examiner indicated the landmarks on 10 reconstructed models.

### Statistics

Statistical analysis was performed in SPSS (v. 26, IBM, New York, USA). Skeletal deformities between the CSP and VSP groups were compared using the χ^2^ test to exclude confounding effects of the patients’ morphology. Intra- and interrater agreement of the measurements were assessed by Bland–Altman-plots [30].

The data were assumed to be non-normally distributed, and the median and interquartile range of the linear and angular measurements were reported. The absolute differences were compared in each experimental group by one-sample Wilcoxon signed rank test to the theoretical value of 0. To compare the accuracy of maxillary positioning between the VSP and CSP groups, the Mann–Whitney U test for independent samples was applied. The correlations between the mean discrepancies in all translational and rotational dimensions were investigated by scatterplots and Spearman’s rank correlation coefficients. Scatterplots were further used to evaluate the correlation between the planned surgical movement and the inaccuracy of maxillary positioning. The global level of significance was set at *p* < 0.05. Individual *p*-values were adjusted by Bonferroni-Holm correction.

## Results

The accuracy of maxillary positioning in orthognathic surgery was investigated in 52 patients and was compared between virtual and conventional surgery planning (a detailed description of the study sample is displayed in Table 2). There was no association between the type of surgery planning and skeletal deformities, regarding skeletal class (p = 0.192), vertical relation (p = 0.498) or maxillary (p = 0.876) and mandibular asymmetry (p = 1.0). Bland–Altman plots revealed high intra- and interrater agreement for all measurements with average differences < 1 mm/ 1° and small limits of agreement.

**Table 2:** Demographic and clinical characteristics of the patient population

Patient population	*N* = 52
CSP group	*n* = 26
Male	*n* = 7
Female	*n* = 19
VSP group	*n* = 26
Male	*n* = 13
Female	*n* = 13
Age of patient population (y)	*M* = 24.4; *SD* = 6.2
Age of CSP group (y)	*M* = 22.9; *SD* = 4.8
Age of VSP group (y)	*M* = 25.9; *SD* = 7.1
Skeletal classes in CSP group	
Skeletal class I (Wits = [− 2; 2 mm])	*n* = *3* (*M* = 0.5; *SD* = 1.1)
Skeletal class II (Wits > 2 mm)	*n* = *4* (*M* = 6.1; *SD* = 3.8)
Skeletal class III (Wits < 2 mm)	*n* = *19* (*M* = − 8.4; *SD* = 4.6)
Skeletal classes in VSP group	
Skeletal class II (Wits > 2 mm)	*n* = *7* (*M* = 3.9; *SD* = 1.9)
Skeletal class III (Wits < 2 mm)	*n* = *19* (*M* = − 8.6; *SD* = 4.5)
Vertical relation in CSP group	
Hyperdivergent (ML-NL > 26.5°)	*n* = *12* (*M* = 34.4; *SD* = 5.0)
Neutral (ML-NL = [20.5; 26.5°])	*n* = *10* (*M* = 25.1; *SD* = 1.2)
Hypodivergent (ML-NL < 20.5°)	*n* = *4* (*M* = 19.0; *SD* = 0.6)
Vertical relation in VSP group	
Hyperdivergent (ML-NL > 26.5°)	*n* = *15* (*M* = 35.2; *SD* = 5.5)
Neutral (ML-NL = [20.5; 26.5°])	*n* = *6* (*M* = 24.2; *SD* = 2.2)
Hypodivergent (ML-NL < 20.5°)	*n* = *5* (*M* = 16.3; *SD* = 2.3)
Asymmetry in CSP group	
Menton deviation > 4 mm	*n* = 10 (*M* = 6.3; *SD* = 2.2)
Mean deviation of A-point from the midsagittal plane (mm)	*M* = 0.1; *SD* = 1.3
Asymmetry in VSP group	
Menton deviation > 4 mm	*n* = 10 (*M* = 7.1; *SD* = 2.3)
Mean deviation of A-point from the midsagittal plane (mm)	*M* = − 0.2; *SD* = 2.2
Surgical intervention in CSP group	
Le Fort I and BSSO	*n* = 22
Le Fort I	*n* = 4
Surgical intervention in VSP group	
Le Fort I and BSSO	*n* = 26

**Fig. 2:**
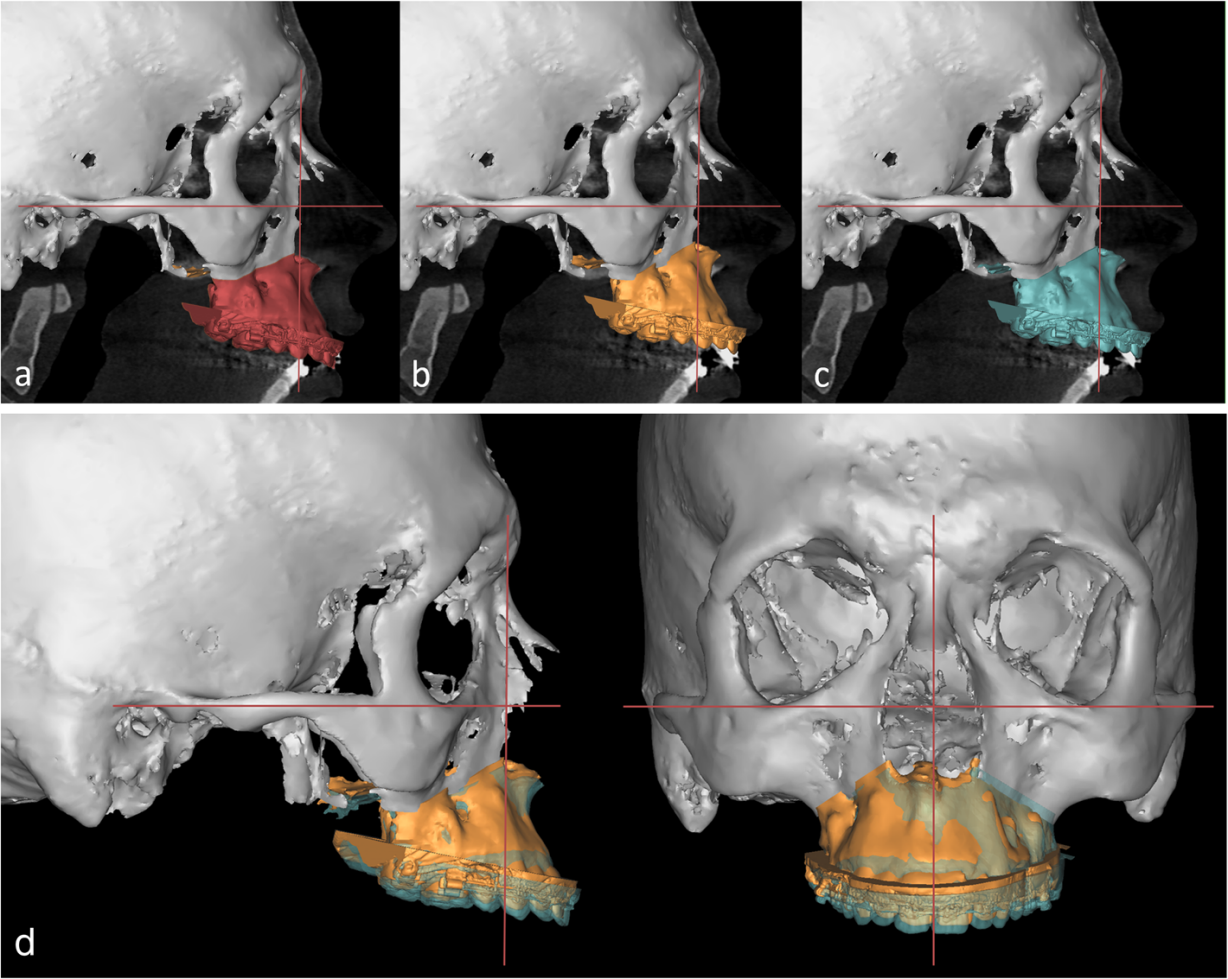
Comparison of the preoperative, the planned and the postoperatively achieved maxillary position: **a** Preoperative CBCT scan with the maxilla in the preoperative position. **b** Preoperative CBCT scan with the maxilla in the planned position. **c** Preoperative CBCT scan with the maxilla in the postoperatively achieved position. **d** The superimposition of the planned (yellow) and the achieved (green) maxillary position demonstrates the commonly observed underimpaction of the maxilla in vertical direction and a mild deviation of the midline in transversal direction

VSP and CSP both demonstrated significant discrepancies between the planned and actual achieved positions of the maxilla (Table 3). Regarding maxillary translation, inaccuracies were highest for vertical movement [i.e., U1(z), see Fig. 2]. The positive value of the mean difference (CSP: 1.4 mm; VSP: 2.1 mm) revealed that this discrepancy was caused by an undercorrection of the planned impactions. For rotational movements, the inclination of the occlusal plane around the y-axis was the least predictable variable with over- and underachievement of the planned maxillary inclination. Considering clinical relevance, in vertical direction 73% in the CSP group and 46% in the VSP group achieved the desired maxillary position (Table 4). Interestingly, in most patients the maxillary position was undercorrected while overcorrection was observed in one case only. No significant difference in accuracy between CSP and VSP was observed in any dimension (Table 5).

**Table 3:** Discrepancy between the planned and achieved maxillary positions in conventional (CSP) and virtual surgery planning (VSP)

Measurement	Mean discrepancy Planned vs. achieved Median (IQR)	Absolute discrepancy Planned vs. achieved Median (IQR)	*p*-value
CSP (n = 26)			
U1 (x) (mm)	− 0.7 (2.3)	1.2 (1.1)	< 0.001*
U1 (y) (mm)	− 0.2 (1.7)	0.7 (1.1)	< 0.001*
U1 (z) (mm)	1.4 (1.6)	1.6 (1.3)	< 0.001*
OcP (x) (°)	0.3 (1.4)	0.9 (1)	< 0.001*
OcP (y) (°)	0.3 (3.3)	1.6 (1.8)	< 0.001*
OcP (z) (°)	− 0.4 (1.5)	0.7 (0.9)	< 0.001*
VSP (n = 26)			
U1 (x) (mm)	− 0.7 (1.6)	1 (1)	< 0.001*
U1 (y) (mm)	− 0.1 (2.7)	1.3 (1.5)	< 0.001*
U1 (z) (mm)	2.1 (2.9)	2.2 (2.2)	< 0.001*
OcP (x) (°)	0.2 (2.0)	1 (1)	< 0.001*
OcP (y) (°)	− 0.2 (3.9)	1.9 (2.7)	< 0.001*
OcP (z) (°)	0.3 (2.0)	0.9 (1.2)	< 0.001*

The mean discrepancy considers whether the maxillary positioning was under- or overcorrected, while the absolute discrepancy describes the total discrepancy regardless of the direction of the positioning error. *P*-values were adjusted by Bonferroni–Holm correction

* Significant

**Table 4:** Success rate and direction of inaccuracy of conventional (CSP) and virtual surgery planning (VSP)

Measurement	Success rate		Direction of inaccuracy	
	Absolute discrepancy < 1 mm or 2° *n* (percentage)	Absolute discrepancy < 2 mm or 4° *n* (percentage)	Under-correction < 2 mm or 4° *n* (percentage)	Over-correction < 2 mm or 4° *n* (percentage)
CSP (n = 26)				
U1 (x)	10 (39%)	23 (89%)	1 (4%)	3 (12%)
U1 (y)	18 (69%)	23 (89%)	2 (8%)	1 (4%)
U1 (z)	10 (39%)	19 (73%)	7 (27%)	0 (0%)
OcP (x)	24 (92%)	26 (100%)	0 (0%)	0 (0%)
OcP (y)	16 (62%)	24 (92%)	0 (0%)	2 (8%)
OcP (z)	24 (92%)	26 (100%)	0 (0%)	0 (0%)
VSP (n = 26)				
U1 (x)	14 (54%)	22 (85%)	3 (12%)	3 (12%)
U1 (y)	11 (42%)	19 (73%)	3 (12%)	4 (15%)
U1 (z)	9 (35%)	12 (46%)	13 (50%)	1 (4%)
OcP (x)	23 (89%)	26 (100%)	0 (0%)	0 (0%)
OcP (y)	14 (54%)	22 (85%)	2 (8%)	2 (8%)
OcP (z)	22 (85%)	26 (100%)	0 (0%)	0 (0%)

Success was defined as absolute discrepancy < 1 mm/ 2° or < 2 mm/ 4° between the planned and the achieved results

**Table 5:** Comparison of the accuracy in maxillary positioning between conventional (CSP) and virtual surgery planning (VSP) based on the absolute discrepancy between the planned and actual postoperative results

Measurement	Average difference in accuracy between CSP and VSP (Median VSP–Median CSP)	*p*-value
U1 (x) (mm)	− 0.2	1 (n.s)
U1 (y) (mm)	0.6	0.162 (n.s)
U1 (z) (mm)	0.6	1 (n.s)
OcP (x) (°)	− 0.1	1 (n.s)
OcP (y) (°)	0.3	0.589 (n.s)
OcP (z) (°)	0.2	1 (n.s)

*p*-values were adjusted by Bonferroni–Holm correction

*n.s.* not significant

**Fig. 3:**
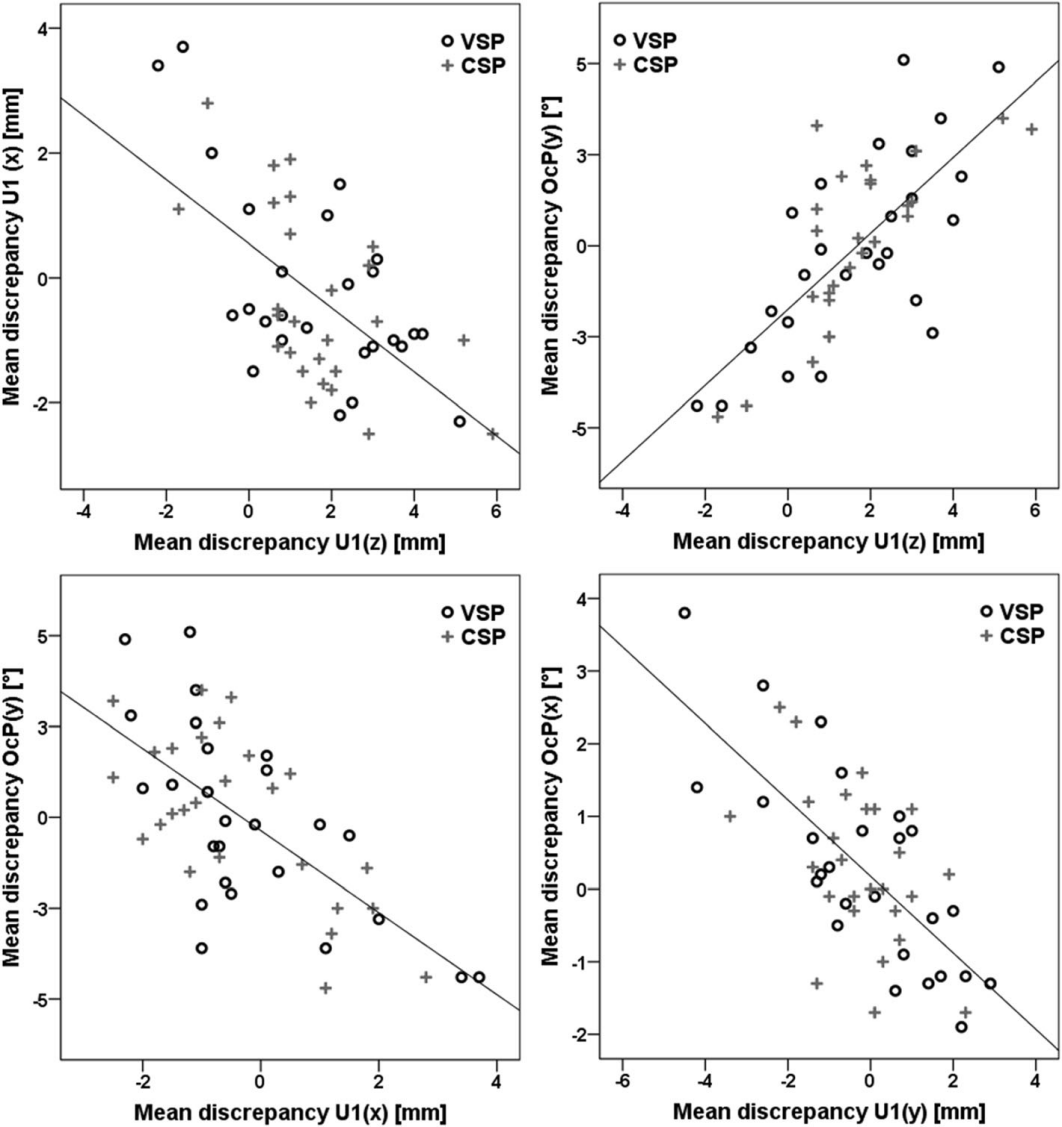
Scatterplots indicated correlations between the mean discrepancies in different translational and rotational movements

The scatterplots (Fig. 3) revealed that inaccuracies in vertical translation correlated significantly with discrepancies in sagittal translation (*r* = − 0.49; *p* < 0.001) and antero-posterior rotation [OcP(y)] (*r* = 0.668; *p* < 0.001). This means that undercorrection of vertical impaction was associated with increased sagittal movement and reduced posterior inclination of the maxilla. Consequently, a correlation between sagittal translation and OcP (y) was found (*r* = − 0.607; *p* < 0.001). Regarding transverse movements, undercorrection of occlusal canting (OcP(x)) correlated with an overachievement in midline correction (*r* = − 0.613; *p* < 0.001).

**Fig. 4:**
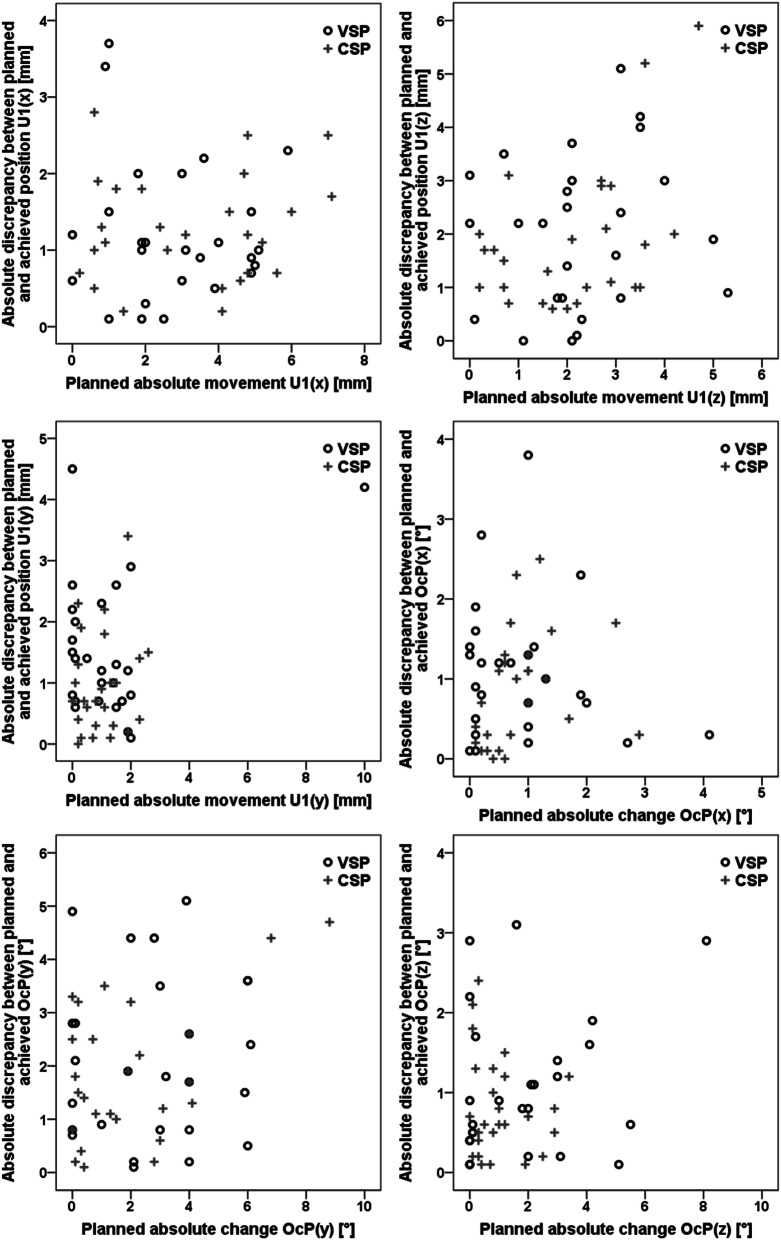
Scatterplots indicated no correlation between the planned surgical movement and the inaccuracy of the result

No correlations between the magnitude of the planned movements and the inaccuracy in maxillary positioning were observed (Fig. 4).

## Discussion

The comparison of VSP and CSP revealed that the clinically achieved predictability of both methods is similar. VSP and CSP demonstrated significant differences between the planned and postoperative outcomes in all dimensions, with the vertical impaction of the maxilla as the least predictable movement. In all but one cases, the vertical movement was undercorrected, which may result in persistence of vertical maxillary excess, increased lower face height and gummy smile. This is not surprising as errors in maxillary impaction are well-known in orthognathic surgery [6, 14, 19, 20, 25] and deviations between the predicted and the actual vertical movement of 10 mm have been described [14].

While the average vertical discrepancy in CSP was within the clinically acceptable limit of 2 mm, the VSP group exceeded this limit by 0.1 mm. However, this difference between both methods was statistically not significant. Nonetheless, this outcome was against our expectations as CSP and VSP result in the same design of surgical splints, which have no control on the vertical maxillary movement. Therefore, the vertical position has to be validated intraoperatively by the use of internal or external reference points [15], what was done by the surgeons in our study in both groups. One possible explanation for this discrepancy may be the differing splint material between VSP and CSP. The VSP material had a lower flexural strength compared to the CSP material, which may have affected the resistance to deformation under the load during surgery. Due to the manufacturing process, the splints in CSP were thicker than in VSP, which possibly had a further effect on splint stability. Additionally, thin splints mask the tendency of the surgeon to remove less bone than required at the superior part of the maxilla. In contrast, a thick, bulky splint reveals a lack of vertical bone removal, especially in the posterior area, more obviously by showing a bony gap in the anterior contact area and encourages the surgeon to remove this interference [31, 32]. However, this contrasts with the expectation that thinner splints are more favorable as they reduce the magnitude of mandibular autorotation and the displacement of the condylar position as seen for example in occlusal registrations, in which accuracy is of tremendous importance [33]. Further studies regarding splint thickness in VSP are required to elucidate this issue. Splint-less surgeries with customized titanium plates and cutting guides eliminate the discussion on splint thickness and provide an interesting option to avoid this vertical error [1].

The direction of surgical movement is believed to affect the outcome of maxillary positioning [34]. However, we observed no correlation between the magnitude of the planned maxillary displacement and the positional discrepancies. Instead, the discrepancies in the sagittal, vertical and transverse dimensions correlated with each other. For example, an undercorrection in the posterior maxillary impaction (i.e., an error in the inclination of the occlusal plane) coincided with increased anterior positioning of the upper central incisors. This interaction between the inclination of the occlusal plane and the antero-posterior position of the incisors is based on geometric reasons and is also observed in errors caused by face-bow transfer in CSP [6, 11]. Against our expectations, this positioning error was also observed in the VSP group. Therefore, we assume that this correlation is caused intraoperatively.

The purpose of this study was not to criticize surgery planning in general, but to emphasize the problems facing the surgeon and the orthodontist: to correct a vertical maxillary excess, occlusal canting or increased gingival display by orthognathic surgery is very demanding, and the type of planning method or splint production does not change that. However, a precautionary overcorrection in surgery planning cannot be recommended, as we observed over- and underachievement in all movements.

The applied analyses of accuracy were chosen very strictly to detect even small positioning errors, and, compared to previous studies [16, 18], avoided reporting only the mean discrepancies. By referring to those summary statistics, similar values on either side of zero cancel each other out and mask the true discrepancies. Instead of evaluating the overall maxillary position [17, 18], our measurements focused on the upper central incisors and the inclination of the occlusal plane. Compared to surface to surface measurements, inaccuracies by this method appear larger [22]. As the patient assesses his/her postoperative smile based on the incisor position, it is important to evaluate the success of maxillary positioning at the incisors.

A further strength of this study lies in the fact that the CSP results were digitized and analyzed with the same method as VSP, which was not done in previous studies. The planned and achieved outcomes were superimposed by a voxel-based algorithm and compared in the same coordinate system. The surgery protocol was consistent for all patients: The condyles were repositioned, and the maxillary surgery was performed first. The patients were not operated on by the same surgeon, even though the same surgeon supervised all surgeries. However, the accuracy between different surgeons in different centers obtained similar results when the same stringent surgery protocol was used [17].

It has to be pointed out that it was beyond the scope of this study to compare the whole planning process between VSP and CSP and that we focused on the errors caused by orientation of the maxillary casts and manufacturing of the splints. For example, unsatisfactory results based on undetected facial asymmetries or incorrect cephalometric diagnosis due to missing 3D information were not part of our investigation. Moreover, the accuracy of mandibular positioning was not investigated because the postoperative CBCTs were taken within 2 weeks of surgery. During this early postoperative period, the possibility that swelling and edema would affect the mandibular position is high, and no reliable analyses could be provided with the intended precision. As a 1 mm error in occlusion might cause more clinical problems than a 1 mm malpositioned maxilla [35], further research investigating the accuracy of mandibular positioning is required.

## Conclusions

In summary, splint-based VSP and CSP result in clinical acceptable accuracy for maxillary positioning. The advantages of VSP, such as the visualization of the preoperative condylar position or the collision of the proximal and distal mandibular segments, as well as the improved communication possibilities between surgeons and orthodontists, provide arguments for the use of VSP. Nevertheless, it should be kept in mind that vertical positioning is challenging in both methods and VSP combined with splint-less procedures should be driven forward to reduce this error.

## Data Availability

The datasets used and/or analyzed during the current study are available from the corresponding author on reasonable request.

## Abbreviations

CSP: Conventional surgery planning
VSP: Virtual surgery planning
CBCT: Cone beam computed tomography
OcP: Occlusal plane
